# That Big Tongue

**Published:** 1897-11

**Authors:** Edward B. Jackson

**Affiliations:** Houston, Texas


### That Big Tongue.

Houston, Texas, July 17th, 1897.

*Editors Texas Medical Journal:*

Referring to the communication by Dr. Thornton, of Plum, we beg to make a few remarks relative to hypertrophic development of the tongue. It is not of such rare occurrence as one might be led to suppose from the trend of the doctor's article. Not unlike many other organs, the tongue is capable, under certain circumstances, of becoming vastly hypertrophied.

We have seen several cases of glossal hypertrophy which were congenital. They were, as is usually the case, met with in children of faulty brain development. They are (the children)

short-lived, and, as a rule, more or less afflicted with idiocy throughout their fleeting existence. When the affliction is not congenital, it is probably the result of some inflammatory process, trauma, or salivation—whether from the use of mercury, gold, or other drugs having the power to produce ptyalism, is an indifferent matter requiring no discussion.

Anything causing and promoting glossitis may be followed by a degree of permanent pressure upon the lymph chains and plugging of the stream currents by inflammatory exudute, infiltrations, and connective tissue growths, which, if sufficiently extensive, may produce obstructive lingual hyperplasia and prolapse, with consequent complete loss of function. When speech is thus entirely impeded, these cases require sectional incision in the shape of a V or U, as the operator may deem most expedient, before any hope of relief can be held out. We have seen perfect return of speech follow the operation upon a physician who, in an unguarded and excited moment, falsely conceived that he was (having at the time an eruption) the victim of syphilis. Acting upon this erroneous idea, he flooded himself with great doses of mercury and potash, increasing the same when his tongue lesion appeared, under the belief that the manifestation was due to paralysis from specific brain disease. His useless specific dosing increased the glossitis until he was compelled to seek relief for complete loss of speech. After the temporary engorgement has subsided, operative measures for the removal of redundant formation is demanded in all cases. It is manifestly the only means of restoring the function of the tongue. Of course, in the instances of children affected with idiocy, the operation promises naught. For cases of acquired lingual hypertrophy we have seen beneficial results in many cases follow the simple removal of an inverted V section from the centre of the tongue.

Edward B. Jackson, M. D.

